# Potential Prognostic Value of Native T1 in Pulmonary Hypertension Patients

**DOI:** 10.3390/life13030775

**Published:** 2023-03-13

**Authors:** John W. Cerne, Christina Shehata, Ann Ragin, Ashitha Pathrose, Manik Veer, Kamal Subedi, Bradley D. Allen, Ryan J. Avery, Michael Markl, James C. Carr

**Affiliations:** 1 Department of Radiology, Feinberg School of Medicine, Northwestern, Chicago, IL 60611, USA; 2 Department of Biomedical Engineering, Northwestern University, Evanston, IL 60208, USA

**Keywords:** pulmonary hypertension, extracellular volume fraction, native T1, late gadolinium enhancement, patient outcomes

## Abstract

Native T1, extracellular volume fraction (ECV), and late gadolinium enhancement (LGE) characterize myocardial tissue and relate to patient prognosis in a variety of diseases, including pulmonary hypertension. The purpose of this study was to evaluate if left ventricle (LV) fibrosis measurements have prognostic value for cardiac outcomes in pulmonary hypertension subgroups. 54 patients with suspected pulmonary hypertension underwent right-heart catheterization and were classified into pulmonary hypertension subgroups: pre-capillary component (PreCompPH) and isolated post-capillary (IpcPH). Cardiac magnetic resonance imaging (MRI) scans were performed with the acquisition of balanced cine steady-state free precession, native T1, and LGE pulse sequences to measure cardiac volumes and myocardial fibrosis. Associations between cardiac events and cardiac MRI measurements were analyzed within PreCompPH and IpcPH patients. IpcPH: LV native T1 was higher in patients who experienced a cardiac event within two years vs. those who did not. In patients with LV native T1 > 1050 ms, the rate of cardiac events was higher. ECV and quantitative LGE did not differ between groups. PreCompPH: native T1, ECV, and quantitative/qualitative LGE did not differ between patients who experienced a cardiac event within two years vs. those who did not. LV native T1 may have potential value for forecasting cardiac events in IpcPH, but not in PreCompPH, patients.

## 1. Introduction

Pulmonary hypertension (PH) is a progressive, life-threatening, and multifactorial disease process that is characterized by an elevated resting mean pulmonary arterial pressure (mPAP) [1,2]. PH is broadly classified into pre-capillary PH (PrePH), isolated post-capillary PH (IpcPH), and combined pre- and post-capillary PH (CPH) based on clinical presentation and hemodynamic measurements [3,4]. Mechanistically, PrePH is defined as pulmonary vascular remodeling associated with an increase in pulmonary vascular resistance (PVR). IpcPH is defined as left-sided heart disease associated with increased pulmonary venous pressures (as measured by pulmonary capillary wedge pressure; PCWP). CPH represents a progression of IpcPH and is defined by concomitant increases in PVR and PCWP. While PrePH, IpcPH, and CPH all lead to right ventricular (RV) failure, elevated PVR heralds RV dysfunction in both PrePH and CPH (collectively termed: Pre-Capillary Component PH [5]; PreCompPH) while incipient left ventricular (LV) dysfunction characterizes the process in IpcPH. PH classification is used to guide optimal treatment and to predict prognosis [6].

Right heart catheterization (RHC) has been the gold-standard diagnostic and prognostic tool for PH. However, because of its invasive nature, there is increasing exploration of alternative prognostic markers in PH patients. In PH patients, cardiopulmonary exercise testing [7] and cardiac MRI measurements [8] have shown correlations with RHC measurements. Cardiac MRI, specifically, has been shown to provide sensitive biomarkers capable of distinguishing PH subgroups [9,10].

Ventricular fibrosis measurements have been shown to provide more sensitive prognoses in patients with LV diastolic dysfunction [11] and systemic sclerosis [12], compared to functional measurements alone. Cardiac MRI can be used to evaluate fibrosis through the detection of increased extracellular space (late gadolinium enhancement, LGE; extracellular volume fraction, ECV) through increased volume measures of extra- and intra-cellular space (native T1). These measures may have clinical value for outcome prediction in patients with cardiac disease [12,13,14,15]. Previous prognostic studies relate to RV functional measurements in PrePH [16,17,18]. 

Given the disparate pathophysiologic processes leading to an elevated pulmonary artery pressure, we hypothesized that LV fibrosis measurements would have prognostic significance in IpcPH, and not in PreCompPH.

## 2. Materials and Methods [10]

### 2.1. Subjects

Approval from the Institutional Review Board (IRB) was obtained, and all subjects provided written informed consent. The initial cohort was identical to that used in a previous study investigating 4D flow-derived velocities in PH patients [8]. Patients with suspected PH who had undergone standard-of-care RHC were identified. Patients with a mPAP ≥ 25 mmHg at rest or mPAP > 30 mmHg during exercise were recruited to undergo a cardiac MRI protocol with venous blood sampling for hematocrit within 28 days of cardiac catheterization. Patients were enrolled between August 2017 and March 2020. Exclusion criteria included: allergy to gadolinium-based contrast agents; severe kidney disease (estimated glomerular filtration rate < 30 mL/min/1.73 m^2^; acute kidney injury; kidney or liver transplant within 8 weeks; any contraindication to MRI (i.e., claustrophobia); pregnant or breastfeeding women; adults unable to provide consent; children; prisoners.

#### 2.1.1. Right Heart Catheterization

Standard-of-care RHC was performed with a 7–9 French sheath via the internal jugular or femoral veins. A Swan-Ganz catheter, connected to an analog pressure recorder, was used to obtain mPAP, systolic PAP, diastolic PAP, pulmonary capillary wedge pressure (PCWP) and right ventricular cardiac output (Q_P_). PVR (in Wood Units (WU)) was calculated using the formula: PVR_RHC_ = ΔP/Q_P_, where ΔP is the trans-pulmonary pressure gradient (ΔP = mPAP − PCWP) and Q_P_ is the flow in the pulmonary artery measured by the Fick principle using the pulmonary artery oxygen saturation.

#### 2.1.2. Classification

PH patients were classified (MV: a cardiologist with 5 years of experience in cardiac imaging) based on a review of their clinical courses, therapeutic histories, and hemodynamic measurements. Patients’ clinical courses and therapeutic histories were considered in addition to hemodynamic measurements, during classification, because invasive parameters are known to vary with a patient’s fluid and metabolic status, and it has been suggested that hemodynamics should be interpreted in the context of the clinical picture [4]. Patients with pulmonary arterial hypertension, pulmonary hypertension due to chronic lung disease, and chronic thromboembolic PH) were considered pre-capillary PH [19]. Patients with pulmonary hypertension due to left heart disease were considered IpcPH or CPH, based on PVR measurements (PVR < 3 WU or PVR ≥ 3, respectively). CPH and PrePH patients were collectively designated as PreCompPH patients.

#### 2.1.3. Clinical Data Collection

The electronic medical record (EMR) was reviewed to determine if a cardiac event occurred after patients’ cardiac MRI scans. Cardiac events were defined as any of the following: heart failure requiring intravenous diuretics; palpitations prompting inpatient observation; or coronary revascularization. If any of these events occurred, the elapsed time between the MRI scan and the event was recorded. Comorbidities (history of smoking, hypertension, hyperlipidemia, diabetes mellitus) and functional status within 4 months of MRI (New York Heart Association (NYHA) functional class, six-minute walk distance, brain natriuretic peptide (BNP), supplemental oxygen use, ascites, peripheral edema) were also retrieved from the EMR (CS: a medical student with 2 years of experience).

### 2.2. Cardiac MRI Data Acquisition [10]

The Cardiac MRI scans were performed by certified technicians using a 1.5 T MRI system (MAGNETOM Aera; Siemens Healthcare, Erlangen, Germany). A three-plane fast localization sequence was used to determine anatomic orientations for subsequent sequences, and four-chamber, two-chamber, and short-axis localizer views were obtained. The pre-contrast portion of the protocol consisted of multiplanar segmented balanced cine steady-state free precession (bSSFP) and native T1 mapping (Modified Look-Locker inversion recovery (MOLLI) technique). After contrast administration (Gadobutrol, 0.1 mL/kg), LGE and MOLLI T1 mapping sequences were performed (10 min and 15 min after contrast, respectively). The combined duration of the MRI sequences was 30 min.

The bSSFP cine acquisition was performed in the two-, three-, four-chamber, and short-axis orientations. Data were acquired during breath holds at end-expiration using retrospective electrocardiogram (ECG) gating. Cine sequence MRI parameters were as follows: field of view (FOV) read = 340 mm × 340 mm, spatial resolution = 1.8 mm × 1.8 mm × 6.0 mm, temporal resolution = 35.49 ms, flip angle = 80°, echo time/repetition time (TE/TR) = 1.16/35.49 ms.

The gradient recalled echo (GRE) bSSFP MOLLI acquisitions were collected pre- and post-contrast (delay = 10 min) with sequence parameters as follows: TE 1.33 ms, flip angle 35°, slice thickness 8.00 mm, pixel size = 1.0 × 1.0 mm^2^, Generalized Auto-calibrating Partial Parallel Acquisition (GRAPPA) with an acceleration factor, R = 2. Imaging reconstruction included the auto-calculation of parametric LV T1 maps.

The LGE pulse sequences were performed with either of two Phase-sensitive Inversion–Recovery (PSIR) pulse sequences: (1) a 2D inversion–recovery (IR) balanced steady-state free precession (bSSFP) pulse sequence (Echo spacing: 2.5 ms, TE 1.05 ms, flip angle 40°, slice thickness 6.0 mm, pixel size = 2.0 × 2.0 mm^2^, GRAPPA with an acceleration factor, R = 2) a segmented 2D IR gradient recalled echo (turboFLASH) pulse sequence (Echo spacing: 8.4 ms, TE 3.25 ms, flip angle 25°, slice thickness 6 mm, pixel size = 1.3 × 1.3 mm^2^, GRAPPA with an acceleration factor, R = 2).

### 2.3. Cardiac MRI Analysis [10]

#### 2.3.1. Volumetric Measurements

**Figure 1:**
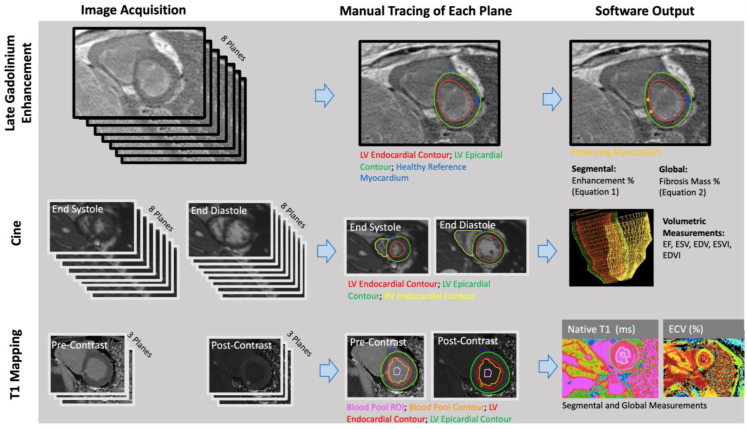
Late gadolinium enhancement (LGE), volumetric, and parametric mapping measurements were obtained with similar workflows. The number of planes obtained and the traced regions differed for each analysis (Reprinted with permission from Ref. [10]. 2022, John W. Cerne).

The RV and LV endocardial borders were manually contoured on short-axis bSSFP cine images at the peak end-diastolic and end-systolic time frames using CVI_42_ (Circle Cardiovascular Imaging, Calgary, AB, Canada) (Figure 1). CVI_42_ generated ejection fractions, end-diastolic volumes, and end-systolic volumes of the right and left ventricles (RVEF, RVEDV, RVESV; LVEF, LVEDV, LVESV). Each patient’s body surface area (BSA) was calculated using the Mosteller equation, and volumes were divided by BSA to get end-systolic and end-diastolic volume indices (RVEDVI, RVESVI, LVEDVI, LVESVI).

#### 2.3.2. Late Gadolinium Enhancement

Quantitative: A semi-automated technique was used to quantify myocardial fibrosis through manual thresholding of the short-axis LGE images on CVI_42_ (Figure 1). A research fellow (JWC: 1 year of experience in cardiothoracic imaging) performed manual tracings of the epicardial and endocardial LV borders. Regions of fibrosis were defined by reference to a manually delineated region of the normal myocardium. Voxels with intensities that were 4 standard deviations above the average intensity of normal myocardium were considered fibrosis. Images were anonymized and analyzed in a blinded fashion and in a random order.

For each scan, global LGE was calculated (Equation (1)) [20]

(1) $$Global\;fibrosis\;mass\;\%=\left(\frac{Mass\;of\;enhancing\;myocardium}{Total\;Myocardial\;Mass}\right)\times 100$$

Qualitative: A radiologist (KS: 2 years of experience in cardiothoracic imaging) performed a qualitative analysis of LGE presence. Patients were determined to have: no LGE; or LGE at one insertional point; or LGE at both insertional points. To evaluate interobserver reliability, a second radiologist (BDA: 4 years of experience in cardiothoracic imaging) assessed the presence and location of LGE in a randomly selected subset of 10 subjects.

#### 2.3.3. Native T1 Mapping

Manual segmentation of T1 maps was performed (AP: a research fellow with 3 years of experience in cardiothoracic imaging) on CVI_42_ (Figure 1). The LV epicardium/endocardium was manually contoured, and regions of interest within the blood pool cavity were demarcated on native t1 and postcontrast images (AP) (Figure 1). The basal, mid, and apical slices from pre- and post-contrast T1 maps were used with patients’ hematocrit values to obtain pixel-wise ECV values (Equation (2)).

(2) $$\lambda =\frac{\frac{1}{T1myocardium\;post\;C}-\frac{1}{T1myocardium\;pre\;C}}{\frac{1}{T1blood\;post\;C}-\frac{1}{T1blood\;pre\;C}}\;ECV=\left(1-Hct\right)\times \lambda$$

Pixel-wise values were converted by the software into average values, on a segment-by-segment basis, for each of the American Heart Association (AHA)-defined myocardial segments. Segmental values were averaged to obtain global ECV and native T1 values for each scan [21].

### 2.4. Statistical Analysis

Descriptive statistics were recorded based on the nature of the compared variables: mean ± standard deviation (continuous variables, normal distribution); median (interquartile range) (continuous variable, non-normal distribution); frequency (percentage) (categorical variables). Shapiro–Wilk test was used to assess continuous variables for normality.

Continuous variables were compared between two groups with independent samples Student’s t-test preceded by Levene’s test (normally distributed) or Mann–Whitney *U* test (non-normally distributed). Chi-square statistics with Yates correction or Fisher’s exact test were used to compare categorical data, depending on the size of the groups being compared (>/=5 and <5, respectively). Kaplan–Meier curve analysis and log-rank test were used to assess cumulative cardiac events.

Statistical analyses were performed with SPSS (IBM corporation, Chicago, IL, USA). All tests were two-tailed with *p* < 0.05 considered statistically significant.

## 3. Results

### 3.1. Patient Characteristics

**Figure 2:**
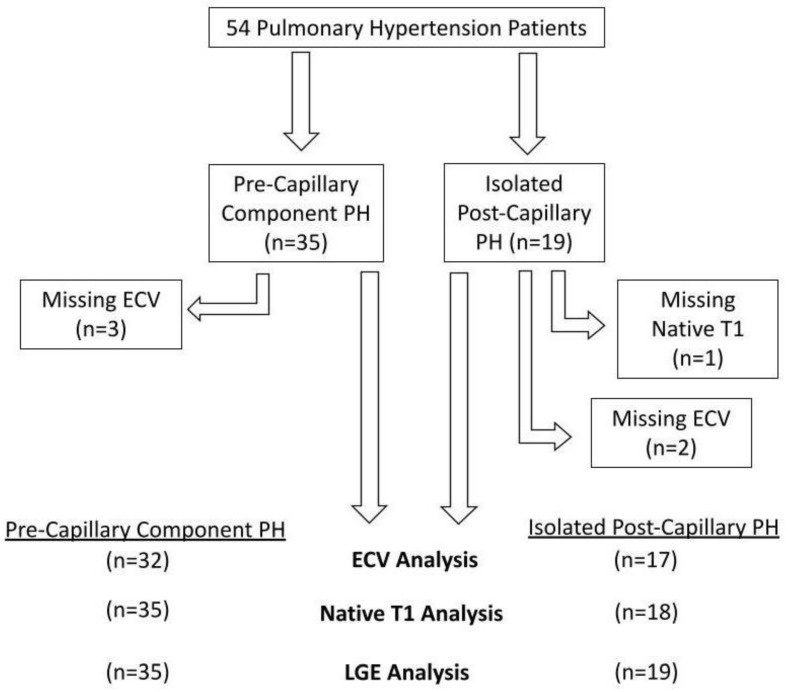
Flow-chart detailing excluded data.

Fifty-four patients were involved in the assessment (35 PreCompPH and 19 IpcPH patients). Patients’ clinical information is depicted in Table 1. Functional status data within 4 months of the MRI scan was not found for all patients: 40/54 patients had a NYHA functional status designation; 12/54 patients had a six-minute walk distance; 30/54 patients had a BNP measurement. There was an inability to calculate ECV in five subjects due to the following reasons: missing post-contrast T1 MOLLI images (n = 2); severe zebra artifact (n = 1); misaligned slice positioning (n = 1); post-contrast dark blood misregistration (n = 1). Native T1 and volumetric indices could not be obtained due to zebra artifact in one subject (n = 1) (Figure 2). Receiver operating curve (ROC) analyses of mPAP and PVR within PreCompPH and within IpcPH patients showed no statistically significant area-under-curve (AUC) values for the occurrence of a cardiac event within 2 years of the cardiac MRI scan (Table 2 and Table 3).

### 3.2. Volumetric Measurements

**Figure 3:**
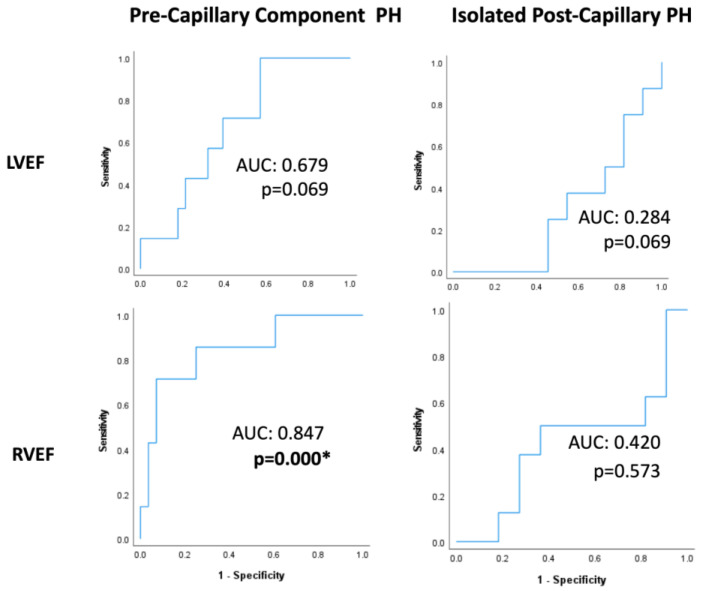
Receiver-operating characteristic curves of left ventricle and right ventricle ejection fractions (LVEFs and RVEFs, respectively) within Pre-Capillary Component and Isolated Post-Capillary pulmonary hypertension (PH) patients. The binary classifier was the occurrence of a cardiac event within 2 years of cardiac MRI. RVEF showed a statistically significant classification ability within Pre-Capillary Component PH patients. * = *p* < 0.05.

ROC analyses within PreCompPH patients showed no statistically significant AUC values for LVEDV (AUC = 0.419; *p* = 0.529), LVESV (AUC = 0.500; *p* = 1.000), LVEDVI (AUC = 0.324; *p* = 0.181), LVESVI (AUC = 0.500; *p* = 0.126), RVEDV (AUC = 0.571; *p* = 0.561), RVESV (AUC = 0.648; *p* = 0.236), or RVESVI (AUC = 0.632; *p* = 0.294) for the occurrence of a cardiac event within 2 years of the cardiac MRI scan. The AUC of LVEF approached significance (AUC = 0.679; *p* = 0.069), and the AUC of RVEF was statistically significant (AUC = 0.847; *p* = 0.000) (Table 2 and Figure 3).

ROC analyses within IpcPH patients showed no statistically significant AUC values for LVEDV (AUC = 0.386; *p* = 0.424), LVESV (AUC = 0.371; *p* = 0.360), LVEDVI (AUC = 0.443; *p* = 0.694), LVESVI (AUC = 0.429; *p* = 0.624), LVEF (AUC = 0.284; *p* = 0.069), RVEDV (AUC = 0.414; *p* = 0.552), RVESV (AUC = 0.371; *p* = 0.371), RVEDVI (AUC = 0.429; *p* = 0.650), RVESVI (AUC = 0.429; *p* = 0.650), or RVEF (AUC = 0.420; *p* = 0.573) (Table 3 and Figure 3).

### 3.3. Late Gadolinium Enhancement

**Figure 4:**
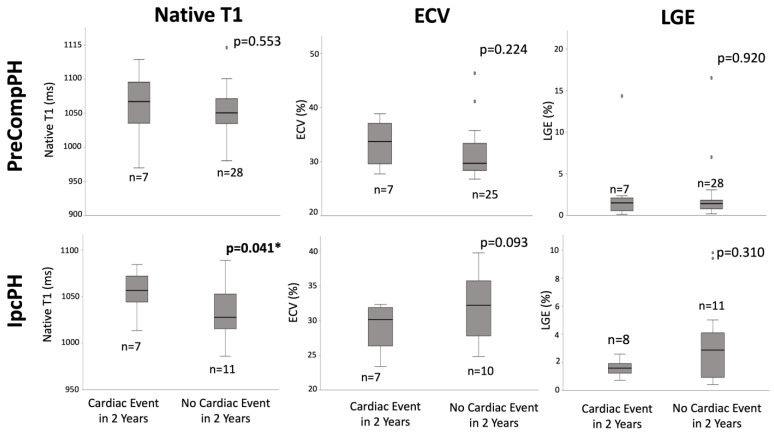
Box-plot comparisons of native T1, extracellular volume fraction (ECV), and late gadolinium enhancement (LGE) between Pre-Capillary Component pulmonary hypertension (PreCompPH) and Isolated Post-Capillary pulmonary hypertension (IpcPH) patients. Mann-U Whitney Tests or Independent Samples *t*-Tests were used based on normality testing by Shapiro–Wilk Test (*p* < 0.05 or *p* > 0.05, respectively). * = *p* < 0.05.

Quantitative: Within PreCompPH patients, there was no statistically significant difference in global fibrosis mass percent between the patients who experienced a cardiac event within two years (1.5 (0, 3.4)%) and those who did not (1.4 (0.2, 2.6)%) (*p* = 0.920). Similarly, within IpcPH patients, there was no statistically significant difference between the patients who experienced a cardiac event within 2 years (1.6 ± 0.6%) and those who did not (2.9 (0, 8.5)%) (Figure 4).

Qualitative: Within PreCompPH patients, the distributions of the numbers of patients with no LGE at insertional points (IPs), LGE at one IP, and LGE at both IPs between patients who experienced a cardiac event within two years vs. those who did not were: 14% vs. 32% (*p* = 0.645); 86% vs. 61% (*p* = 0.380); and 0% vs. 7% (*p* = 1.000), respectively. Within IpcPH patients, the distributions of the numbers of patients with no LGE at IPs, LGE at one IP, and LGE at both IPs between patients who experienced a cardiac event within two years vs. those who did not were: 63% vs. 64% (*p* = 1.000); 13% vs. 36% (*p* = 0.338); 25% vs. 0% (*p* = 0.164) (Table 4).

### 3.4. Native T1

**Figure 5:**
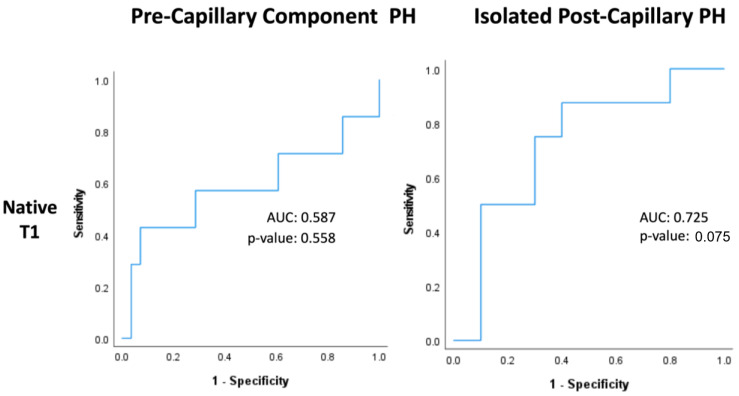
Receiver-operating characteristic curves of native T1 within Pre-Capillary Component and Isolated Post-Capillary pulmonary hypertension (PH) patients. The binary classifier was the occurrence of a cardiac event within 2 years of cardiac MRI.

**Figure 6:**
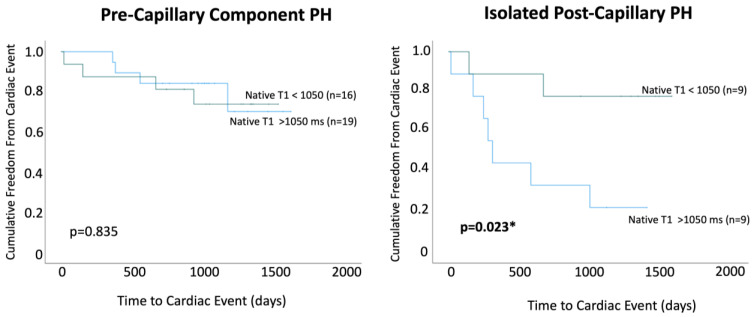
Kaplan–Meier survival curves were used to compare patients with native T1 values < 1050 ms and >1050 ms within Pre-Capillary Component pulmonary hypertension (PH) and Isolated Post-Capillary pulmonary hypertension (PH) patient groups. * = *p* < 0.05.

Within PreCompPH patients, the amount of LV native T1 in patients who experienced a cardiac event within two years was higher than the amount of native T1 in patients who did not experience a cardiac event within two years (1061.0 ± 53.4 ms vs. 1049.2 ± 33.9 ms), though without statistical significance (*p* = 0.553). ROC analysis showed an AUC of 0.587 for the association between LV native T1 and the occurrence of a cardiac event within two years of cardiac MRI (*p* = 0.558) (Figure 5). Kaplan–Meier survival analysis showed no statistically significant survival difference between patients with a LV native T1 <1050 ms (n = 16) and patients with a LV native T1 >1050 ms (n = 19) (*p* = 0.835) (Figure 6).

Within IpcPH patients, there was a statistically significant higher amount of LV native T1 in patients who experienced a cardiac event within two years of cardiac MRI (1055.7 ± 24.2 ms vs. 1032.1 ± 28.9 ms) (*p* = 0.041) (Figure 4). ROC analysis showed an AUC of 0.725 for the association between LV native T1 and the occurrence of a cardiac event within two years of cardiac MRI (*p* = 0.075) (Figure 5). Kaplan–Meier survival analysis showed a statistically significant lower rate of survival in patients with LV native T1 >1050 ms (n = 9) compared to patients with LV Native T1 < 1050 ms (n = 9) (*p* = 0.023) (Figure 6).

### 3.5. Extracellular Volume Fraction

Within PreCompPH patients, there was no statistically significant difference in global LV ECV percent between the patients who experienced a cardiac event within two years of cardiac MRI (33.4 ± 4.4%) and those who did not (29.7 (24.4, 35.0)%) (*p* = 0.224) (Figure 4). ROC analysis showed an AUC of 0.632 for the association between LV ECV and the occurrence of a cardiac event within two years of cardiac MRI (*p* = 0.273).

Within IpcPH patients, there was no statistically significant difference in global LV ECV percent between the patients who experienced a cardiac event within two years of cardiac MRI (28.9 ± 3.5%) and those who did not (32.0 ± 5.1%) (*p* = 0.093). ROC analysis showed an AUC of 0.314 for the association between LV ECV and the occurrence of a cardiac event within two years of cardiac MRI (*p* = 0.168).

## 4. Discussion

The presented results show that fibrosis and volumetric assessments may be of value for the prediction of cardiac outcomes in PH patients, but this value may depend on whether a patient’s PH is marked by elevated PVR (and subsequent PreCompPH characterization). Volumetric and hemodynamic measurements appear less promising.

The results of this study show that LV native T1 measurements may be able to forecast cardiac events in IpcPH patients, but not in PreCompPH patients. No association was found between LGE or ECV and the occurrence of a cardiac event in either subset of PH patients. Though several fibrosis quantification techniques have been simultaneously studied for their potential to prognosticate muscular dystrophy patients [21], amyloidosis patients [22], and a heterogeneous group of patients referred for cardiac MRI [23], our study is the first study to simultaneously assess the relationship between prognosis and all three fibrosis measurement techniques in a PH cohort. In a retrospective cohort study of 223 patients with PAH, Saunders et al. [24] found that septal T1, RV insertion point T1, and LV free-wall T1 were not associated with mortality. In this study, 59 patients died during a follow-up period of 29 months which allowed for univariate Cox proportional hazards regression analysis. The present study differed as native T1 values were assessed by an LV global summary measurement; however, global native T1 of the LV has been previously shown to correlate with insertional point T1 (r = 0.75; *p* < 0.05) [25]. The results of our study are similar to the results of Saunders et al. as no outcome association was observed in PreCompPH (of which PAH is a subtype). Yet our results suggest that this may not be the case in IpcPH patients. Previous studies have reported a predictive relationship between native T1 measurements in outcomes. These works have studied all-comer patients [26,27], patients with diabetes [28], and non-ischemic dilated cardiomyopathy patients [29]. We believe these cohorts are more akin to IpcPH given the collective absence of elevated PVR (and likely absence of fulminant right heart failure) in these patient populations, in which right ventricular function has less impact on prognosis.

Our results showing a lack of statistically significant associations between LGE, ECV, and cardiac outcomes are consistent with the previous literature. In a study using native T1, ECV, and LGE measurement techniques in the non-infarcted myocardium of patients with coronary heart disease, native T1 and ECV were independent predictors of outcome [29]. Both tissue characterization techniques surpassed LGE assessment, and native T1 performed better than ECV. It was suggested that this may be due to effects other than fibrosis, such as inflammation, which affect native T1 values more than ECV. Our results suggest that this effect may not be limited to coronary artery disease patients, and that pathophysiological processes outside of fibrosis may be occurring in the LV of IpcPH patients. Furthermore, in a group of hypertrophic cardiomyopathy patients, 30% of LGE-negative segments showed an elevated native T1 time [30], suggesting that native T1 is a more sensitive biomarker than LGE. ECV, native T1, and LGE all purport to measure myocardial fibrosis, but they achieve this through surrogate fibrosis measurements. Native T1 appears most relatable to the outcome and may therefore be the best means of fibrosis approximation.

It has also become appreciated that RV functional measures assessed by cardiac MRI are associated with a functional state, exercise capacity, and survival in patients with PAH (a subset of PreCompPH) [31,32,33,34]. RVEF and RVESV have been shown capable of distinguishing PH severity grades [31], and the loss of RV function is associated with a poor outcome irrespective of any changes in PVR [34]. Whether RV functional measures continue to hold clinical value in IpcPH patients has been under-investigated. We indeed found that a lower RVEF was characteristic of PreCompPH patients who went on to experience a cardiac event in two years, but not of IpcPH experiencing a cardiac event in two years.

Interestingly, invasive measurements were not prognostic in IpcPH or PreCompPH patients. Elevated pulmonary artery pressures have previously been shown to be associated with outcomes in patients with left heart disease and PH [35]. PVR measurements greater than 2.2 Wood Units portend a worse prognosis in all subgroups of PH [36]. Previous works showing the relationship between hemodynamic measurements have focused on all-cause mortality [36,37,38] or heart-failure-specific hospitalizations [36], singularly. These assessments were not possible in our cohort, considering the small number of observed deaths and hospitalizations secondary to heart failure. The present study defined outcomes as cardiac events because previous associations between fibrosis measurements and cardiac outcomes have been observed in heart transplant patients [13]. In our small cohort, the inclusion of ‘cardiac event’ outcomes extending to palpitations and coronary revascularization was necessary to investigate the relationship between outcomes and several cardiac MRI parameters.

## 5. Limitations [8,10]

There are several limitations to our study. First, the small sample size of the present cohort, combined with a low death rate (6 out of 54 patients) during the follow-up period, precluded the use of regression and survival analyses in our study. Larger studies are needed to allow for robust statistical testing of our preliminary findings. Second, up to 28 days elapsed between cardiac MRI and RHC. Disease progression or remission may have occurred during this time. For this reason, patients were classified by a cardiology fellow with experience following PH patients. RHC values, volumetric measurements, and clinical factors were all considered during the classification process. Spruijt et al. [39] similarly classified idiopathic PAH, systemic scleroderma-related PH, and CTEPH all as PrePH-type, without reference to RHC measurements. It would have been ideal for RHC and cardiac MRI to have been collected close together, as has previously been done [40], but this was not possible given the recruitment protocol. This study is further limited by the recruitment protocol, as the mPAP threshold used to define PH has recently changed (in 2015, a resting mPAP >/= 25 mmHg defined PH [4]; in 2022, a resting mPAP of >20 mmHg defined PH [41]), with new guidelines established by the European Society of Cardiology and the European Respiratory Society. Our study used the older guidelines, as the recruitment protocol occurred during this time. It has been suggested that mPAP values used in isolation cannot characterize the PH clinical condition and do not define the pathological process per se [19]. Although our recruitment protocol may have been a more sensitive protocol for recruiting PH patients, the higher mPAP threshold precluded the inclusion of PH patients with milder diseases. Our conclusions may not hold relevance in these newly defined PH patients, with a resting mPAP between 20 and 25 mmHg.

## 6. Conclusions

This preliminary proof-of-concept study demonstrates that native T1 measurements of the LV may be able to forecast the occurrence of cardiac events in IpcPH patients, and that this ability does not persist in PreCompPH patients. RVEF does not appear to hold prognostic value in IpcPH patients but may be prognostic in PreCompPH patients.

## Figures and Tables

**Table 1:** Patient characteristics.

	Pre-Capillary Component PH			Isolated Post-Capillary PH		
Variables	Cardiac Event within 2 Years (n = 7)	No Cardiac Event within 2 Years (n = 28)	*p*-Value	Cardiac Event within 2 Years (n = 8)	No Cardiac Event within 2 Years (n = 11)	*p*-Value
**Comorbidities**						
HLD	3 (43%)	10 (36%)	1.000	3 (38%)	5 (45%)	1.000
Smoking	5 (71%)	10 (36%)	0.112	5 (63%)	6 (54%)	1.000
Diabetes	2 (29%)	8 (29%)	1.000	2 (25%)	4 (36%)	1.000
HTN	4 (57%)	15 (54%)	1.000	7 (88%)	9 (82%)	1.000
**Functional Status**						
NYHA Functional Class	-	2.5 (1.5, 3.5)	-	3 ± 0.7	3 (1, 2)	0.343
Six-Minute Walk Distance	-	364 ± 57	-		429 ± 4.2 (only 2 values)	-
BNP (pg/mL)	-	193 ± 176	-	122 ± 72	515 ± 200	0.016 *
**Clinical Status**						
Supplemental Oxygen	3 (43%)	12 (43%)	1.000	0	0	1.000
Peripheral Edema	3 (43%)	9 (32%)	0.670	4 (50%)	7 (64%)	0.658
Ascites	0	2 (7%)	1.000	1 (13%)	2 (18%)	1.000

PH: pulmonary hypertension, HLD: hyperlipidemia, HTN: hypertension, BNP: brain natriuretic peptide, * = *p* < 0.05.

**Table 2:** ROC analysis for the occurrence of a cardiac event within 2 years of cardiac MRI scan in pre-capillary component PH.

Variables	Area Under Curve (AUC)	*p*-Value
**Fibrosis Measures**		
Native T1	0.587	0.558
ECV	0.632	0.273
LGE	0.478	0.876
**LV Volumetric Measures**		
LVEDV	0.418	0.529
LVESV	0.500	1.000
LVEDVI	0.324	0.181
LVESVI	0.500	0.126
LVEF	0.679	0.069
**RV Volumetric Measures**		
RVEDV	0.571	0.561
RVESV	0.648	0.236
RVEDVI	0.478	0.859
RVESVI	0.632	0.294
RVEF	0.847	0.000 *
**Hemodynamic Measures**		
mPAP	0.445	0.129
PVR	0.511	0.108

PH: pulmonary hypertension, ECV: extracellular volume fraction, LGE: late gadolinium enhancement, * = *p* < 0.05.

**Table 3:** ROC analysis for the occurrence of a cardiac event within 2 years of cardiac MRI scan in isolated post-capillary PH.

Variables	Area Under Curve (AUC)	*p*-Value
**Fibrosis Measures**		
Native T1	0.725	0.075
ECV	0.314	0.168
LGE	0.371	0.385
**LV Volumetric Measures**		
LVEDV	0.386	0.424
LVESV	0.371	0.360
LVEDVI	0.443	0.694
LVESVI	0.429	0.624
LVEF	0.284	0.069
**RV Volumetric Measures**		
RVEDV	0.414	0.552
RVESV	0.371	0.371
RVEDVI	0.429	0.643
RVESVI	0.429	0.650
RVEF	0.420	0.573
**Hemodynamic Measures**		
mPAP	0.300	0.119
PVR	0.529	0.847

PH: pulmonary hypertension, ECV: extracellular volume fraction, LGE: late gadolinium enhancement.

**Table 4:** Qualitative LGE analysis.

Variables	Cardiac Event Occurred within 2 Years	No Cardiac Event Occurred within 2 Years	*p*-Value
**Pre-Capillary Component PH**	(n = 7)	(n = 28)	
LGE at No IPs	1 (14%)	9 (32%)	0.645
LGE at One IP	6 (86%)	17 (61%)	0.380
LGE at Both IPs	0	2 (7%)	1.000
**Isolated Post-Capillary PH**	(n = 8)	(n = 11)	
LGE at No IPs	5 (63%)	7 (64%)	1.000
LGE at One IP	1 (13%)	4 (36%)	0.338
LGE at Both IPs	2 (25%)	0	0.164

PH: pulmonary hypertension, LGE: late gadolinium enhancement, IP: insertional point.

## Data Availability

The data presented in this study are available on request from the corresponding author.
